# Computational analysis of prolyl hydroxylase domain-containing protein 2 (PHD2) mutations promoting polycythemia insurgence in humans

**DOI:** 10.1038/srep18716

**Published:** 2016-01-12

**Authors:** Giovanni Minervini, Federica Quaglia, Silvio CE Tosatto

**Affiliations:** 1 Department of Biomedical Sciences and CRIBI Biotechnology Center, University of Padova, Viale G. Colombo 3, 35121, Padova, Italy; 2 CNR Institute of Neuroscience, Viale G. Colombo 3, 35121, Padova, Italy

## Abstract

Idiopathic erythrocytosis is a rare disease characterized by an increase in red blood cell mass due to mutations in proteins of the oxygen-sensing pathway, such as prolyl hydroxylase 2 (PHD2). Here, we present a bioinformatics investigation of the pathological effect of twelve PHD2 mutations related to polycythemia insurgence. We show that few mutations impair the PHD2 catalytic site, while most localize to non-enzymatic regions. We also found that most mutations do not overlap the substrate recognition site, suggesting a novel PHD2 binding interface. After a structural analysis of both binding partners, we suggest that this novel interface is responsible for PHD2 interaction with the LIMD1 tumor suppressor.

Polycythemia is a rare condition, where a large number of different mutations promote a pathological increase in red cells^1^. Typically, two major classifications are commonly accepted. Primary polycythemia (*polycythemia vera*) is promoted by the deregulation of the Janus Kinase 2 (JAK2) gene^2^. Secondary erythrocytosis is due to a more complex genetic environment, e.g. Chuvash polycythemia^3^. The annual incidence rate varies from 0.02 to 44/100,000 inhabitants^3^,^4^,^5^,^6^, depending on different populations and types of polycythemia. In the literature, the polycythemic trait was reported in patients harboring mutations in hypoxia-inducible factor 1α (HIF-1α), von Hippel-Lindau protein (pVHL) as well as in hypoxia-inducible factor prolyl hydroxylase 2 (PHD2) (formally also known as Egl nine homolog 1, EGLN1)^7^. Indeed, it was demonstrated that mutations of almost all major proteins involved in the oxygen-sensing pathway are related and/or causative of secondary erythrocytosis^7^. Furthermore, while mutations affecting both pVHL and HIF-1α share a concomitant erythropoietin hormone (EPO) deregulation^1^, EPO production is generally unaffected for PHD2 mutants^8^, suggesting a different molecular mechanism for this class of mutants. Two different scenarios were thus derived, with a primary polycythemia characterized by low EPO serum concentrations and erythroid progenitors hyper-responsive to EPO stimulation, whereas in secondary polycythemia erythroid progenitors conserve a normal response to EPO^9^. PHD2 is a member of a small family composed of three proteins (termed PHD-1 to -3) sharing the same fold^10^ but presenting different activity and specialization^11^,^12^. PHD2 is constitutively expressed in most tissue types^13^,^14^ while PHD1 and PHD3 present a narrower expression profile^15^ limited to specific organs (e.g. testis). PHDs require oxygen and 2-oxyglutarate as substrates and Fe (II) and ascorbate as cofactors, catalyzing the oxidation of highly conserved HIF-1α proline residues^16^ and acting as the real oxygen sensors in cells. PHD2 contains 426 residues and is structurally composed of a long intrinsically disorded N-terminal region (residues 1–187) and a well structured oxygenase domain which represents the real catalytic centre^17^ (residues 188–418). In a recent study, Ladroue *et al.*^18^ proposed a two way classification for the pathogenic risk related to PHD2 mutations, with class I addressing mutations promoting a weak HIF-1α deficiency and class II, mostly malignant and predisposing to polycythemia and cancer development. In their study, the authors experimentally identified seven PHD2 mutations found in secondary erythrocytosis patients. Some, e.g. PHD2 D254H and H374R, localize in or close to the enzymatic site, impairing PHD2 activity. In contrast, both the PHD2 P200Q and R371H mutants do not affect HIF-1α regulation, suggesting a non-HIF-1α related activity of PHD2, at least in humans^18^. PHD2 activity is enhanced by physical interaction with the scaffold protein LIMD1 to form a multi-protein complex yielding efficient HIF-1α degradation^19^. LIMD1 is a tumor suppressor member of the Ajuba family^20^, which participates in the assembly of numerous protein complexes (i.e. cell-cell adhesion, differentiation and proliferation). The protein contains three consecutive LIM domains at the C-terminal region, which mediate protein-protein interactions and are preceded by an intrinsically disordered pre-LIM N-terminal proline/serine-rich region^21^,^22^. Experiments conducted by Sharp and coworker^19^ mapped the PHD2 internal binding sites into the pre-LIM region within residues 186–260. The same experiments also showed that LIMD1 interacts with PHD1 and PHD3^19^, the other members of the human PHD protein family. PHD3 is a shorter protein lacking the long disordered N-terminal domain of PHD2 (also present in PHD1). This finding is particularly relevant, as it demonstrates that interaction with LIMD1 is mediated by the globular PHD domain. Although this early finding appeared promising, molecular details driving erythrocytosis insurgence remain poorly understood. In particular, the lack of erythrocytosis-related mutations in the human PHD1 and PHD3^23^ limit the comprehension of the general PHD properties. To further assess the potential relationship between erythrocytosis, cancer outcome and HIF-1α deregulation, we characterized twelve PHD2 mutations found in polycythemic patients through a bioinformatics approach. Mutations were collected from the literature and then mapped on the PHD2 structure. A combination of sequence analysis and stability predictors was used to investigate the pathogenicity risk of each mutation. *In silico* disease mutation correlation was extensively assessed over the last years^24^,^25^, with information about disease-causing protein alterations identified and collected in large mutation databases, e.g. dbNSFP^26^ and GWAS^27^. Our analysis showed that polycythemia causative mutations mostly affect a region not directly involved in PHD2 enzymatic activity, but rather tend to localize on a restricted area and may form a previously undescribed protein-protein interaction interface.

## Methods

### Sequence feature characterization

The PHD2 (isoform 1) and LIMD1 sequences (accession code: Q9GZT9 and Q9UGP4, respectively) were downloaded from Uniprot^28^. Homologous sequences for both proteins were retrieved and selected from OMA Browser^29^ using standard parameters and visualized using Jalview^30^. Multiple sequence alignments (MSA) for PHD2 and LIMD1 were built with Clustal Omega^31^ and used as input for conservation analysis with Consurf^32^. A conservation score was calculated with the Scorecons^33^ server, using default parameters. Secondary structure prediction was performed with PSIPRED^34^, while prediction of intrinsic disorder was performed using C-Spritz^35^. MobiDB^36^ was used to compare disorder predictions obtained for the human PHD2 with PHD orthologs. The presence of linear motifs was investigated using the ELM server^37^. The sequence logo for LIMD1 was built with WebLogo^38^ and interacting residues were predicted with Anchor^39^.

### Structural analysis

Three-dimensional structure investigation was performed by visual inspection using two deposited human PHD2 crystal structures (PDB code: 3HQU and 3HQR, respectively^17^). Quantitative measurements (i.e. atom distances, solvent accessibility area, torsion angles, residues that engage in hydrogen and disulfide bonds) of interacting residues were calculated with RING^40^. The two crystal structures were chosen as they present the enzyme with the catalytic β2β3 loop in open and closed conformation^17^ (active and inactive state, respectively) and in complex with a peptide representing the HIF-1α CODD region (residues 549–582) substrate. Sequence conservation was mapped onto the three-dimensional structure using Consurf^32^. The mutant models for eight mutations were built with ClustAlign^41^ and HOMER (http://protein.bio.unipd.it/homer/). GROMACS 4.6.5^42^ was used for 1,000 steps of steepest descent minimization to relax the mutant structures. The PHD2 interacting domain was selected as reported in^19^. A LIMD1 three-dimensional structure was predicted *ab initio* with Rosetta^43^ using an optimized protocol for intrinsically disordered proteins^44^. The quality of proposed PHD2 mutant models, as well as LIMD1 structures, were evaluated with FRST^45^ and QMEANclust^46^, while molecular structures were visualized with Chimera^47^. Mutations affecting charged residues were investigated with BLUUES^48^. Networks of interacting residues of wild type and mutant PHD2 were generated with RING^40^ and compared to highlight interactions perturbed by mutations.

### Mutation analysis and correlation with human diseases

Eight computational methods were used to predict the stability change and impact of nsSNPs on protein function. Pathogenicity predictions were carried out using SNAP^49^, I-Mutant^50^, Polyphen^51^, Pmut^52^, SNPs3D^53^, FoldX^54^, Eris^55^ and NeEMO^56^. Since computational methods have moderate accuracy^57^, different methods such as support vector machines or structure based predictors were used to avoid overlaps in stability prediction. We calculated a prediction score based on a number of methods that define a mutant as deleterious, presence of functional structural elements as well as considering the conservation. We then combined conservation, stability prediction and computational data to assess whether a PHD2 mutant was pathogenic, neutral or ambiguous. Only mutants for which at least three lines of evidence were available (e.g. >6 predictors, conservation and structural analysis) were classified as likely pathogenic or pathogenic (Table 1). Mutants for which predictors and conservation analysis disagreed (e.g. <6 predictors) were assigned as ambiguous. Finally, neutral was only assigned to mutants classified as neutral by all used methods. A panel of 12 missense mutations found in the literature and previously associated with polycythemia was used to investigate the pathogenic effect on PHD2. These are: V138A^58^, Q157H^18^, P165S^58^, P200Q^18^, N203K^59^, D254H^18^, K291I^59^, P317R^60^, R371H^18^, H374R^61^, R398X^18^ and K423E^59^. Correlation with human diseases (i.e. cancer) was investigated with the Structure-PPi^62^ and dSysMap^63^ tools.

## Results

### Sequence Analysis of Missense Mutants

**Figure 1:**
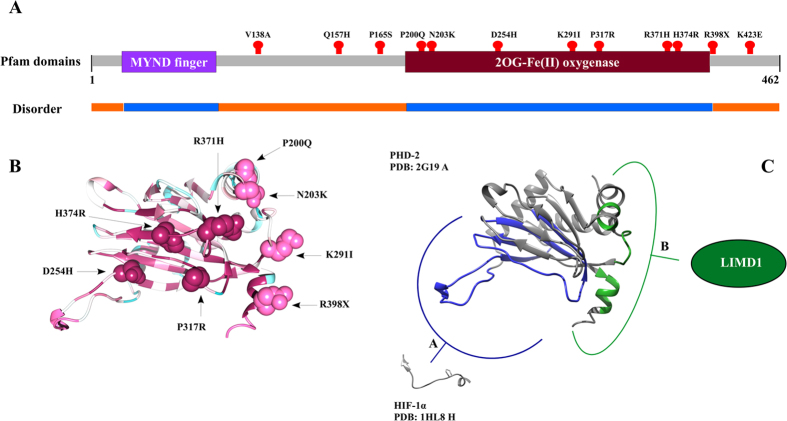
Overview of PHD2 structural analysis. (**A**) A simplification of human PHD2 functional domain organization is shown as a colored bar with grey representing the intrinsically unfolded regions. Purple was used for the MYND finger domain, a region considered important for the interaction with proteins of the HSP90 pathway, including p23^74^. Dark red represents the catalytic relevant oxygenase domain. Red dots on the protein sequence highlight the position of mutations characterized in this work. Predicted disorder is shown as an orange line and predicted structure in light blue. (**B**) The PHD2 structure with the β2β3 loop in open conformation (PDB code: 3HQU) is shown as cartoon, where mutated PHD2 residues are shown as spheres and the degree of conservation is mapped on the structure from magenta (highly conserved) to cyan (unconserved). (**C**) Representation of PHD2 interacting surfaces. In blue, the catalytic site and surrounding area involved in substrate recognition. In green, the novel putative LIMD1 binding interface where we found mutations yielding secondary erythrocytosis.

Following our previous work on PHD mutations in cancer^10^, we identified from the literature twelve human PHD2 mutants described in polycythemic patients (Table 1) and weakly correlated with cancer outcome. One of them, R398X, inserts a stop codon generating a shorter protein lacking 64 residues at the C-terminal region. Intrinsic disorder prediction shows that this region is constitutively unfolded, while prediction with ELM^37^ shows a putative PDZ domain binding motif between residues 419–426 (Fig. 1A). The finding suggests that polycythemia observed in patients with this mutation may be promoted by a regulative function/mechanism lost upon deletion. Conservation analysis was conducted to address the pathogenic effect of the other mutants. Eight mutations (Q157H, P200Q, N203K, D254H, P317R, R371H, H374R and K423E) affect highly conserved positions, suggesting a functional role for these residues (Figure S1). Indeed, P200Q and N203K are located in a region required for nuclear import^64^, while D254H affects the catalytically relevant β2β3 loop. The loop is known to regulate the substrate specificity of PHD enzymes^17^, suggesting a potential effect on PHD2 catalytic activity. P317R, R371H and H374R are localized inside the dioxygenase domain. The structured domain is well conserved among species (Fig. 1B) and we suspect that mutations in this position may severely destabilize the protein fold. K423 is a conserved residue in the intrinsically disordered region close to the C-terminus. Due to its sequence conservation we believe that mutation K423E may interfere with protein-protein interactions engaged by the C-terminal tail. Experimental validation of this result would further confirm a functional role for the PHD2 C-terminus.

### Mutations assessment and stability predictions

Of the twelve mutations described as promoting polycythemia insurgence in the literature, H374R is frequently found in patients with both polycythemia and paraganglioma^61^, a rare neuroendocrine tumor^65^,^66^. While their correlation with polycythemia is clear, the molecular details driving the pathological effects remain not completely understood. The combined application of eight different *in silico* predictors with conservation and structural analyses was chosen as a strategy to obtain the most reliable prediction of their functional impact (Table 1). While most mutations seem to promote a pathogenic effect through protein destabilization, V138A, P165S, Q157H mutants were classified as likely neutral. These mutations localize in a vast intrinsically disordered region (Fig. 1A) and we believe that they only induce small structural rearrangements, sufficiently tolerated by this flexible region. Q157H was further found in patients sharing a polycythemic phenotype with normal EPO levels^18^, suggesting normal PHD2 enzymatic activity for this specific mutant and reinforcing the prediction of neutral behavior. P200Q was predicted as ambiguous, as stability predictors tend to classify the mutation as stabilizing, while pathogenic predictors predicted a possibly damaging effect. N203K was predicted as likely pathogenic with 5/8 predictors in agreement. Both P200 and N203 are conserved in sequence and a structural investigation highlighted a putative nuclear import motif in this region (Figure S1). No inconsistency was obtained for D254H and the mutation affects a region in the catalytically relevant β2β3 loop. As the region is clearly exposed to the solvent, we believe that the predicted neutral behavior is mainly due to a compatible substitution between hydrophilic amino acids, albeit exposing opposed charges. We classified the mutation as ambiguous, as the β2β3 loop is known to be important for substrate recognition^16^, allowing highly selective HIF-1α binding. We believe the resulting negative to positive charge inversion to be potentially able to reduce the PHD2 binding stability and reduce catalytic activity. K291I, P317R, R371H, H374R were predicted as pathological, with mutations affecting the structured domain of PHD2. We then performed a “neighbor analysis” of residues mapping on PHD2 crystal structures to better assess the pathological effect of these specific mutations in vicinity of known functional residues. We found that four mutations are physically close to residues relevant for cancer progression (Table 2). In particular, amino acids N203, D254, H374 are close to residues mutated in human colon tissue adenocarcinoma and small cell carcinomas of lung and bladder.

### Structural analysis of PHD2 mutated residues

**Figure 2:**
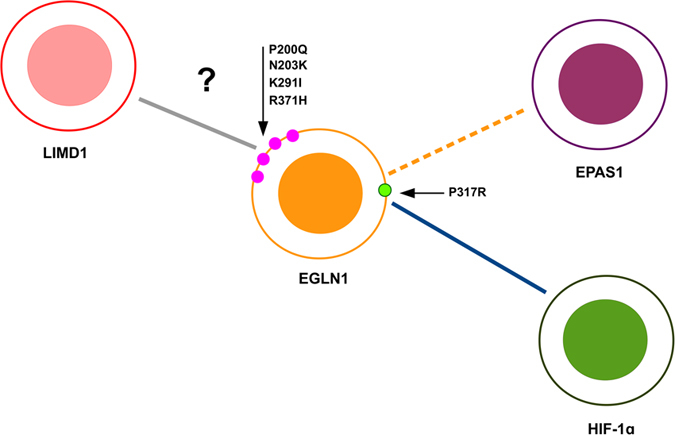
Protein-protein interaction map generated with dSysMap. The network represents interacting proteins affected by mutations and related to polycythemia development. Blue edges represent experimentally validated interaction, while a dotted line was used to describe interactions derived from structural similarity. A grey line is for validated interactions with lack of molecular details. The small circles represent mutations affecting the PHD2 globular domain, with light green representing the mutation P317R known in the literature to affect the interaction with HIF-1α. Magenta circles are the four amino acid that we predict to form the PHD2-LIMD1 binding interface.

A structural characterization was conducted on seven mutants in order to explain their structural impact. PHD2 is a large protein, presenting a vast and intrinsically disordered N-terminal region. We addressed this issue by limiting the investigation to amino acid positions for which experimental data was available (i.e. PDB codes: 3HQU and 3HQR^17^). Indeed, the two crystal structures represent a structural change involving the flexible β2β3 loop which likely occurs concomitant with HIF1α binding^17^. The P200Q mutation localizes on the so-called α1 helix of PHD2. The wild type proline is exposed to the solvent and close to Cys201 which is covalently bound to Cys208. Mutation of Pro200 may reduce helix α1 stability and promote the loss of interaction between Cys201 and Cys208. No relevant structural effect was predicted for the N203K mutant. Indeed, the wild type glutamine is exposed to the solvent, apparently not forming relevant interactions with surrounding residues. The region is predicted by ELM^37^ as important for a linear motif which regulates the nuclear import of PHD2. We believe that the pathological effect may be enhanced by lack of nuclear import. Residues Lys243 and Asp254 are placed in the β2β3 loop and the pathogenic effect is related to variation in catalytic loop flexibility. Asp254 is a key residue for PHD2, coordinating the 2-oxyglutarate inside the catalytic centre. Mutation D254H abolishes this interaction and reduces catalytic activity. The residue Lys291 is located on a solvent exposed loop connecting the strands β4 and β5, in a curved region close to helix α4. Substitution with isoleucine (mutant K291I) may promote local misfolding and a reduction in protein stability due to the hydrophobic behavior of this residue. Analysis shows that the P317R mutation is placed in a loop connecting the β4- and β5-sheets, where wild type proline confers structural rigidity and allows D315 to coordinate the iron ion inside the catalytic site. Substitution with arginine introduces a repulsive effect with R370 placed in front of P317 (Fig. 1B). We believe that repulsion between positively charged residues may yield functional impairment and severe local unfolding. A different pathogenic effect was predicted for H374R. This histidine forms the catalytic site coordinating the iron ion required for the catalysis. Although ariginine is known to coordinate ions as well, we believe that steric hindrance may reduce the catalytic activity of this mutant. Of note, mutations N203K, D254H, K291I, R371H affect exposed amino acids localized in a restricted area forming what we consider a putative polar binding surface (Fig. 1C). Notably, the region does not overlap with the HIF-1α binding area. Thus, mutations in this area should not interfere with HIF-1α regulation mediated by PHD2. The finding may explain the lack of HIF-1α deregulation observed in patients with this mutation^18^. In order to validate the hypothesis of a novel protein-protein interaction surface, we mapped disease-related missense mutations on the PHD2 crystal structure. Analysis with dSysMap^63^ shows mutation P317R interfering with HIF-1α binding and the result is coherent with data in the literature describing the mutation as causative of polycythemia^60^. This mutation is also predicted as relevant for the interaction with EPAS1 (Endothelial PAS domain-containing protein 1), also known as HIF-2α (Hypoxia-inducible factor-2alpha), a transcription factor involved in the induction of oxygen regulated genes and, when mutated, of erythrocytosis insurgence^67^. The analysis also shows that the solvent-exposed area covering residues P200, N203, K291 and R371 is apparently not involved in already known protein-protein interactions (Fig. 2). Very recently, Sharp and coworkers showed that PHD2 and pVHL form a ternary complex with the LIMD1 (LIM domain-containing protein 1) scaffold protein^19^. The authors demonstrated that LIMD1 increases HIF-1α degradation rate by acting as a physical scaffold, binding simultaneously PHD2 and pVHL to form an enzymatic niche where HIF-1α is rapidly hydroxylated by PHD2 and degraded by pVHL^20^. We therefore reason that specific mutations characterized in this study may prevent PHD2/LIMD1 association, resulting in unregulated red cell over-production, potentially as part of the adaptive response to PHD2–LIMD1–VHL complex loss.

### Structural LIMD1 characterization

**Figure 3:**
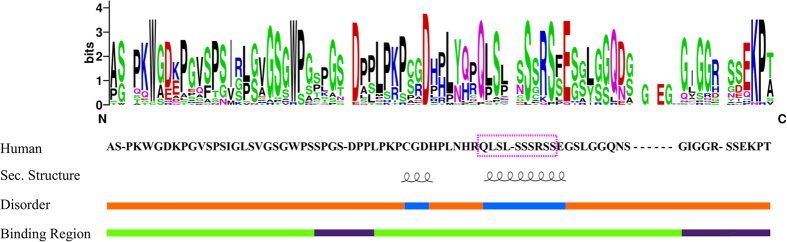
Overview of the LIMD1 fragment features. The LIMD1 sequence logo of the entire PHD2 binding domain is shown with the human LIMD1 sequence (residues 186–260) and predicted disorder below. Predicted disorder is shown as an orange line and predicted structured segments in light blue. Predicted short α-helices are above and predicted binding regions forming structural segments are presented in dark purple against a light green background. The multiple sequence alignment used to build this logo is presented in Supplementary file 1. A magenta box highlights the motif containing the phosphorylable Ser233 found in this LIMD1 segment.

**Figure 4:**
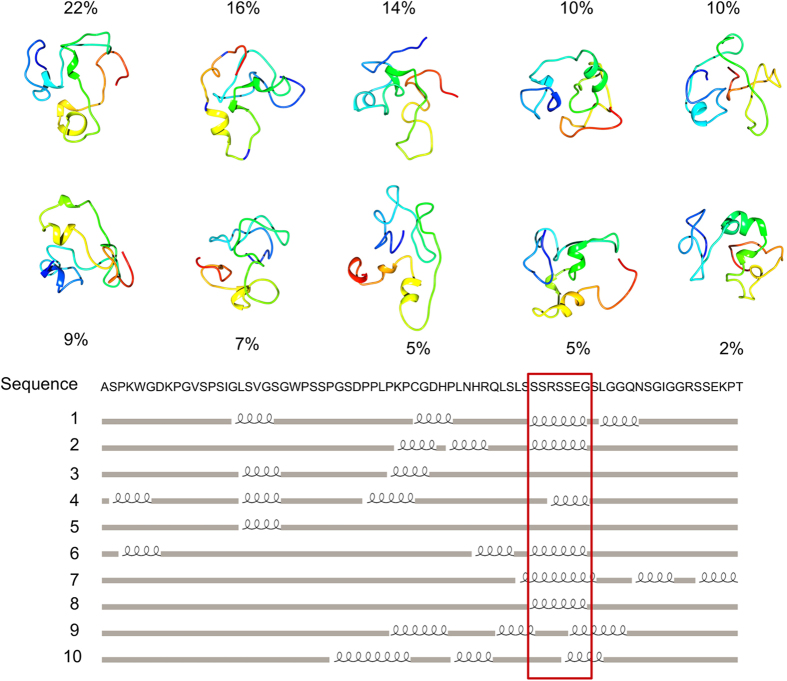
Ribbon models of LIMD1 fragment residues 186–260. The best ten predicted models are shown, ordered by relative frequency. Three-dimensional structures were predicted *ab initio* with Rosetta and extracted by clustering 25,000 decoys. The LIMD1 secondary structures, calculated starting from the three-dimensional models, are shown together with the sequence. A red box is used to highlight the structured elements in agreement between models.

We suggest that the putative interaction interface surrounding residues P200, N203, K291 and R371 acts as a LIMD1 binding area (Fig. 1C), with erythrocytic pathological phenotypes resulting from a reduction of the LIMD1 and PHD2 interaction. To test this hypothesis, we decided to characterize the LIMD1 PHD2-interacting domain. Intrinsic disorder prediction shows this region to be constitutively unfolded and lacking a fixed structure. In particular, we found the amino acid signature of long disorder for residues 196–312, mostly overlapping the PHD2 binding site (Fig. 3). This finding suggests that a disordered region drives PHD2/LIMD1 interaction. We then predicted the three-dimensional structure to highlight structural elements reinforcing this hypothesis. Since no homologous crystal structures were available for LIMD1, we based our prediction on *ab initio* methodology, using a disorder specific optimization protocol^44^. The ten best ranking models (Fig. 4) were then used for further structural investigation. As expected, the three-dimensional structures that resulted are mainly disordered. Nevertheless, short and transient α-helices were predicted in all models (Fig. 4) between residues 223–244. Short secondary structure elements are common in disordered proteins, frequently overlapping functionally relevant regions^68^. We therefore reasoned that a conservation analysis should suggest specific residues driving the PHD2/LIMD1 interaction. A web logo of the PHD2 binding domain constructed from a sequence alignment of LIMD1 orthologs is presented in Fig. 3. The entire LIMD1 fragment is conserved in sequence, showing a high concentration of polar residues at the fragment center and three negative amino acids. A recent large scale experiment demonstrated that Ser233 could be phosphorylated^69^. Considering our results, we believe that the modification may be relevant to regulate the PHD2/LIMD1 interaction. Our previous analysis on PHD2 suggested that residues P200, N203, K291, R371, R398 may form a novel polar binding surface. Coupling this finding with LIMD1 characterization, we suggest that LIMD1 residues within the 216–242 interval may be the molecular determinant of PHD2/LIMD1 interaction.

## Discussion

In the literature, mutations affecting the PHD2, HIF-1 and pVHL genes were correlated with cancer insurgence^70^ and familiar polycythemia^61^,^71^, a syndrome characterized by an over-production of red blood cells. Currently, HIF-1 and pVHL are well known to directly regulate the expression of several genes involved in oxygen homeostasis, angiogenesis as well as oxidative metabolism regulation^72^, where PHD2 acts as primary sensor of oxygen concentration^73^. Mutations promoting PHD2 deregulation are well known in the literature, mostly affecting the catalytic site or reducing enzymatic activity^16^. On the other hand, PHD2 mutations affecting non-catalytic regions were correlated with polycythemia disease insurgence^18^. We used a bioinformatics approach to investigate the pathological effect of twelve different PHD2 mutations. Structural characterization and pathogenicity prediction confirmed that only few mutations promote a direct disruptive effect on the catalytic site, while a part of them seems to localize on less relevant regions of PHD2. We found that four polycythemia-related mutations describe a putative alternative binding area on the PHD2 surface. This area does not overlap the HIF-1 binding interface, suggesting a PHD2 regulative function. Indeed, polycythemia patients are frequently characterized by increased red cell production with normal EPO levels. In particular, the condition is prevalent in PHD2 mutated patients^18^, where HIF-1α degradation is conserved, albeit reduced. In the literature, a macromolecular interaction between PHD2 and LIMD1 was described, with the latter acting as a physical enhancer of PHD2 activity^19^. Mutations abolishing PHD2/LIMD1 interaction should not interfere with PHD2 mediated HIF-1α hydroxylation, rather they may reduce the turnover efficiency yielding a pathological phenotype. The PHD2 binding domain is localized in a LIMD1 region known as pre-LIM, mostly disordered and characterized by low identity with other proteins. To investigate the molecular details driving PHD2/LIMD1 interaction, we modeled the binding fragment using an *ab initio* strategy. The region appeared disordered as expected, with transient short α-helix elements. We also found that the partially structured element corresponds to a polar and well conserved region, with three highly conserved negative residues. Our PHD2 structural analysis suggested that residues P200, N203, K291, R371 form a polar binding surface. Coupling these findings, we believe that LIMD1 residues within the 216–242 interval are the molecular determinant of PHD2/LIMD1 interaction. Due to the *in silico* nature of our analysis, an experimental validation of the obtained results should be carried out. Nevertheless, our results suggest that PHD2 specialization may go beyond the sole catalytic activity. In other words, these mutations reinforce the idea of a PHD2 enzyme acting as gene regulator, where ternary interactions with other molecular partners, such as LIMD1, may act as signal transducer of a finer hypoxia response regulation.

## Additional Information

**How to cite this article**: Minervini, G. *et al.* Computational analysis of prolyl hydroxylase domain-containing protein 2 (PHD2) mutations promoting polycythemia insurgence in humans. *Sci. Rep.* **6**, 18716; doi: 10.1038/srep18716 (2016).

## Supplementary Material

Supplementary Information

## Figures and Tables

**Table 1:** Results by different computational methods used to explain possible stability change, protein aberration and local unfolding of human PHD2.

Variant	SNAP	Pmut	SNPS3D	I-Mutant3.0	PolyPhen	Eris	FoldX	NeEMO	Conservation	PathogenicityPrediction	PredictedStructural Effect	Phenotype
V138A	Neutral	Neutral	Tolerated 1.10	Increase 5	Benign 0.012	NA	NA	NA	Variable	Likely Neutral	NA	Erythr. Sec.
P165S	Neutral	Neutral	Tolerated 1.23	Increase 8	Benign 0002	NA	NA	NA	Variable	Likely Neutral	NA	Erythr. Sec.
Q157H	Neutral	Neutral	Tolerated 1.44	Increase 6	Benign 0.289	NA	NA	NA	Variable	Likely Neutral	NA	Erythr. Sec. Normal EPO
K423E	Neutral	Neutral	Deleterious −2.72	Decrease 7	Probably damaging 0.999	NA	NA	NA	Conserved	Likely Pathogenic	NA	Erythr. Sec.
P200Q	Neutral	Pathological	Deleterious −0.42	Decrease 8	Possibly damaging 0.952	ΔΔG −4.46	ΔΔG −2.60	ΔΔG −0.02	Conserved	Ambiguous	Disturbs the Cys201-Cys208 interaction	Erythr. Sec. High EPO
N203K	Neutral	Pathological	Tolerated 0.44	Decrease 2	Benign 0.032	ΔΔG 3.61	ΔΔG 1.06	ΔΔG 0.65	Conserved	Likely Pathogenic	Disturbs a putative linear motif	Polycyth. Vera Low EPO
D254H	Non-Neutral	Pathological	Deleterious −3.68	Decrease 8	Possibly damaging 0.885	ΔΔG 4.09	ΔΔG 3.75	ΔΔG 1.48	Conserved	Likely Pathogenic	Reduces β2β3-loop stability	Erythr. Sec. Normal EPO
K291I	Neutral	Pathological	Tolerated 0.62	Decrease 6	Possibly damaging 0.736	ΔΔG −1.53	ΔΔG −0.69	ΔΔG −0.48	Variable	Likely Pathogenic	Reduces protein stability	Familial history of Erythr. Sec.
P317R	Non-Neutral	Pathological	Deleterious −2.28	Decrease 8	Probably damaging 0.999	ΔΔG −3.17	ΔΔG 1.66	ΔΔG 0.45	Conserved	Pathogenic	Reduces catalytic site stability	Erythr. Sec. Normal EPO
R371H	Non-Neutral	Pathological	Deleterious −2.14	Decrease 9	Probably damaging 0.997	ΔΔG −5.66	ΔΔG 2.45	ΔΔG 2.23	Conserved	Pathogenic	Reduces protein stability	Modest erythr. Normal EPO
H374R	Non-Neutral	Pathological	Deleterious −3.02	Decrease 6	Probably damaging 0.999	ΔΔG 3.25	ΔΔG 3.05	ΔΔG −0.70	Conserved	Pathogenic	Impairs iron ion coordination	Erythr. Sec. Para-aortic paraganglioma
R398X	NA	NA	NA	NA	NA	NA	NA	NA	NA	Ambiguous	Inserts a STOP codon	

Conservation is derived from ConSurf, which classifies each residue as variable (values 1–3), average (values 4–6), or conserved (values 7–9). Pathogenicity prediction for missense mutants was obtained comparing the results from eight different methods (I-Mutant, Polyphen, SNPs3D, Pmut, SNAP, Eris, NeEMO and FoldX) with conservation analysis and structural investigation. A missense mutant was classified as deleterious when more than six out of ten lines of evidence predict it as deleterious. One mutant was classified ambiguous since most of the methods fail to get a result, while structural investigation suggests a catalytic role. Surface mutants are predicted to alter the two interfaces (A and B) of PHD2, which have roles, respectively, in HIF-1α complex formation (experimentally validated) and interaction with LIMD1 tumor suppressor (predicted). n/d, not determined.

**Table 2:** Neighbor residues analysis performed with Structure-PPi^62^.

Variant	Neighbor residue	Primary site	Primary histology	Histology subtype	PubMedidentifier
P200Q	—	—	—	—	—
N203K	202	Urinary tract	Bladder	N. S.	21822268
D254H	—	—	—	—	—
K291I	292	Large intestine	Colon	Adenocarcinoma	22895193
P317R	—	—	—	—	—
R371H	—	—	—	—	—
H374R	344	Lung	N.S.	Small cell carcinoma	22941189
R398X	—	—	—	—	—

Vicinity relevant positions are derived from COSMIC^75^.
