# Supplementary material for: Computational analysis of prolyl hydroxylase domain-containing protein 2 (PHD2) mutations promoting polycythemia insurgence in humans
Source: Sci Rep. 2016 Jan 12;6:18716. doi: 10.1038/srep18716 (PMC4709589; doi:10.1038/srep18716)
Supplement: Supplementary Information [file srep18716-s1.pdf]

Computational analysis of prolyl hydroxylase domain-containing protein 2 (PHD2) mutations promoting polycythemia insurgence in human

Giovanni Minervini[1], Federica Quaglia[1], Silvio CE Tosatto[1, 2*].

*[1]Department of Biomedical Sciences and CRIBI Biotechnology Center, University of Padova, Italy. [2]CNR Institute of Neuroscience, Padova, Italy *Corresponding Author*

Supplementary Material

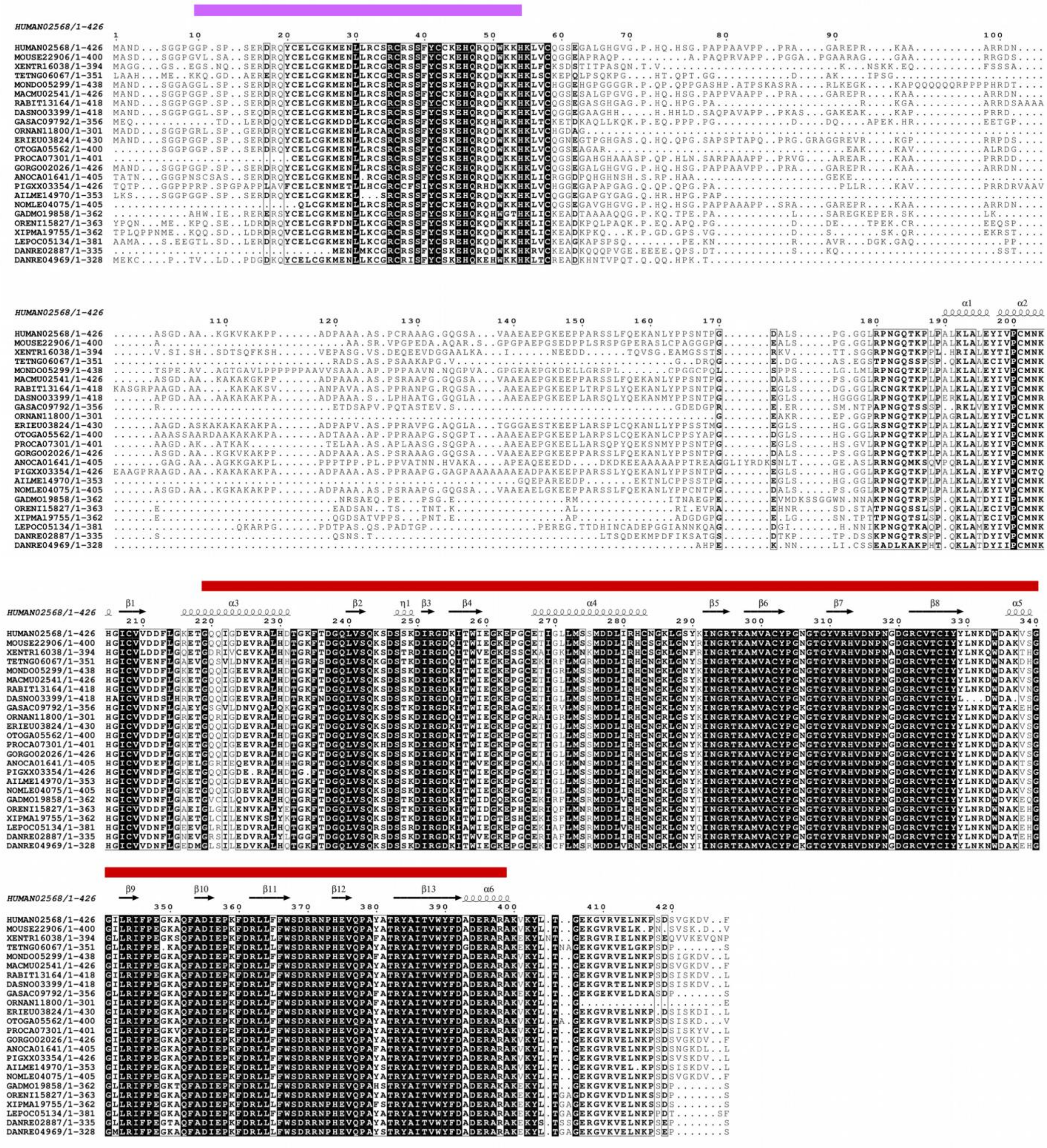

Figure S1. PHD2 sequence features. Domain architecture and protein sequence alignment of PHD2. Functional domains are shown on the top part. Purple highlights the MYND zinc finger domain, required for nuclear import, and dark red is used for oxygenase domain. Secondary structure and homologous PHD2 sequences are shown in the center. The picture was generated with Espript[1].

References

1. Gouet, P., Robert, X. & Courcelle, E. ESPript/ENDscript: Extracting and rendering sequence and 3D information from atomic structures of proteins. *Nucleic Acids Res.* **31,** 3320ï 3323 (2003).
